# Traumatic brain injury and post-injury sleep fragmentation differentially alter the microglial transcriptome

**DOI:** 10.3389/fimmu.2025.1689773

**Published:** 2026-01-05

**Authors:** Morgan A. Taylor, Rebecca Boland, Samuel Houle, Zoe M. Tapp, Amara C. Davis, Christopher Cotter, John F. Sheridan, Jonathan Godbout, Olga N. Kokiko-Cochran

**Affiliations:** 1 Department of Neuroscience, College of Medicine, The Ohio State University, Columbus, OH, United States; 2 Institute for Behavioral Medicine Research, The Ohio State University, Columbus, OH, United States; 3 Chronic Brain Injury Program, The Ohio State University, Columbus, OH, United States

**Keywords:** TBI, sleep fragmentation, neuroinflammation, microglia, monocytes, RNA-sequencing

## Abstract

**Introduction:**

Traumatic brain injury (TBI) is a global source of injury-related death and disability, and survivors often suffer functional and psychiatric consequences that persist for years. Neuroinflammation, mediated in part by microglia, perpetuates chronic dysfunction after TBI and leaves survivors vulnerable to the effects of secondary immune challenges. Previous data from our lab shows that 30 days of mechanical sleep fragmentation (SF) aggravates microglia- associated neuroinflammation in C57BL/6 mice, impairing recovery after TBI.

**Methods:**

To better understand the mechanisms through which microglia contribute to impairment following post-TBI SF, we used flow cytometry to analyze multiple cell types from brain and peripheral tissues of C57BL/6 mice who received a TBI or sham injury followed by 7 or 30 days of SF or control housing. Next, bulk RNA sequencing was used to analyze gene expression in microglia and coronal slice from the ipsilateral brain. We analyzed differentially expressed genes (DEGs) within each tissue type to determine how ipsilateral brain and microglia are independently influenced by TBI and SF. We also compared microglial DEGS directly to those of coronal slice, gaining novel insight into how microglia contribute to dysfunction in the ipsilateral brain after TBI and post-injury SF.

**Results:**

Flow cytometry revealed transient increases in monocyte infiltration to the brain 7 days post-injury (DPI) that resolved by 30 DPI. SF did not exacerbate the immune response to injury within peripheral tissues or the brain at either of these time points. From our transcriptomic analysis, we identified distinct sets of DEGs which are uniquely dysregulated by TBI, SF, and the combination of TBI and SF. Notably, we found distinct subsets of olfactory genes that are differentially dysregulated by TBI and SF in the ipsilateral brain, as well as significant enrichment of cell-cell communication and steroidogenesis pathways that are specifically disrupted in microglia compared to the rest of the brain.

**Discussion:**

Through in-depth transcriptional analysis we identify potential molecular targets that shed light on the mechanisms of TBI-induced microglial activity and reveal how SF after TBI alters this response. Together, these data could inform therapeutic strategies that target neuroinflammation to improve chronic recovery after brain injury.

## 1 Introduction

Traumatic brain injury (TBI) is a major source of injury-related death and disability, affecting more than 20 million individuals every year (1). Medical advances have led to a decline in TBI-related deaths, but this contributes to a growing population of survivors who suffer chronic injury-related consequences that can persist for several years after injury. Many of these chronic issues are perpetuated by neuroinflammation and mediated by microglia (2–4). As the resident immune cells in the brain, microglia are critical in maintaining homeostasis. After TBI, microglia mount a rapid immune response, releasing cytokines and chemokines and initiating important mechanisms of repair (5, 6). Some populations of reactive microglia persist long after the initial injury response, continuing to express cytokines and perpetuating a heightened proinflammatory environment in the brain (7). This can lead to more tissue damage and impaired functional recovery as inflammation persists (8). Reactive microglia contribute to the recruitment of peripheral immune cells, further exacerbating the inflammatory environment in the brain (9). Work from our group shows that some microglia can also transition to a primed state, characterized by increased inflammatory markers such as MCHII and CD68 (7). Primed microglia mount an exaggerated inflammatory response to subsequent immune challenge, which can exacerbate cognitive deficits and further impair recovery (10). Experimental evidence demonstrates that forced turnover of microglia after TBI alleviates behavioral and cognitive deficits, highlighting the role of primed microglia in exacerbating post-injury outcomes (11, 12). Altogether, these results pinpoint microglia as a key player in the neuroimmune response to brain injury.

Prolonged neuroinflammatory damage that perpetuates during the chronic phase of injury provokes lingering dysfunction of stress signaling (13–16). This leaves survivors susceptible to the negative consequences of post-injury stressors that stimulate an immune response. Importantly, TBI rarely occurs in isolation. Many environmental stimuli, such as those experienced by TBI survivors during recovery in a hospital or rehabilitation setting, result in sleep loss (17). However, the neuroimmune effects of environmental disturbances after TBI are not well defined. To study the influence of environmental stimuli that modify stress signaling and sleep, we developed a model of post-TBI sleep disruption using mechanical sleep fragmentation (SF) in adult mice (18–20). We previously reported that 7 and 30 days of post-TBI SF aggravates TBI-induced microglia reactivity and neuroinflammation in stress responsive brain regions, resulting in hippocampal-dependent cognitive deficits (19). Yet, the combined and independent effects of TBI and SF on peripheral immune cells and microglia at this chronic post-injury time point remained unclear. TBI results in blood-brain-barrier disruption and increased release of inflammatory cytokines and chemokines, both of which facilitate infiltration of peripheral immune cells that may distinctly shape the neuroimmune environment (21, 22). Here, we complete a comprehensive analysis of the peripheral immune response to TBI and SF using flow cytometry 7 and 30 days post-injury (DPI). We also define the transcriptional signature of microglia and other brain cells using bulk RNA sequencing 30 DPI. Our goal was to define how TBI and post-TBI SF influence the neuroimmune landscape, both independently and together. We hypothesized that 30 days of post-injury SF would have a robust impact on gene expression compared to TBI alone, and that observed changes in the brain would be due to resident, central immune cells rather than infiltrating peripheral immune cells. Flow cytometry revealed transient increases in monocyte infiltration 7 DPI which resolved by 30 DPI. SF did not exacerbate injury-induced responses within peripheral tissues or the brain at either of these timepoints. Through our transcriptional analysis, we identified distinct immune-related genes that are differentially influenced by TBI and SF in the ipsilateral brain and in microglia. Importantly, we make direct comparisons between microglial and brain gene expression, informing how TBI- and SF-induced microglial changes contribute to changes in the ipsilateral hemisphere as a whole. Of note, distinct olfaction and steroidogenesis pathways are differentially dysregulated by TBI and SF, and multiple cell-cell communication pathway genes are specifically disrupted in microglia after post-TBI SF compared to TBI alone. Overall, these findings are an important step in understanding TBI-induced microglial activity and characterizing how post-TBI stressors, such as sleep disruption, influence the neuroimmune response.

## 2 Materials and methods

### 2.1 Mice

For all experiments, equal numbers of 8–10 week-old male and female C57BL/6 mice were obtained from Charles River Laboratories (Wilmington, MA). Mice were separated by sex and group-housed in The Ohio State University Lab Animal Resource (ULAR) facilities under conditions approved by the Institutional Animal Care and Use Committee (IACUC) and in accordance with the NIH guidelines for the Care and Use of Laboratory Mice. Mice were provided *ad libitum* food and water access and were housed in a 12h:12h light/dark cycle (lights on 6AM-6PM).

### 2.2 Surgery and lateral fluid percussion injury

Established procedures were used for surgery and injury (19, 23, 24). For surgical preparation, anesthesia was induced with 4% isoflurane gas, then maintained at 2% isoflurane for the duration of the procedure. A 3mm craniectomy was trephined on the right parietal bone, midway between bregma and lambda, to expose the intact dura mater. A modified polypropylene needle hub with an internal diameter of 3mm was fixed to the skull using super glue and stabilized with dental acrylic. After surgery, mice recovered in their home cages. The following day, anesthesia was induced using 4% isoflurane gas for 4 minutes. For mice receiving a TBI, the hub was attached to the FPI device, and a fluid pulse measuring 1-1.2 atm was delivered onto the exposed dura mater. The hub was then removed and the incision was closed. Sham mice were similarly anesthetized and had hubs removed, but they did not receive FPI. All animals recovered on a heating pad and latency to self-righting reflex was recorded as a measure of injury severity.

### 2.3 Sleep Fragmentation

After injury, half of all mice were housed in SF chambers (Lafayette Instruments), where a sweeper bar moved across the cage bottom every 2 minutes daily from 6AM-10AM to mechanically disrupt sleep at the beginning of the light cycle as previously described (19, 25). SF occurred every post-injury day 6AM-10AM until tissue collection at 7 or 30 DPI. Control mice were housed in the same room but were not exposed to mechanical SF. Mice were provided food and water *ad libitum* and a 12h:12h light/dark cycle (lights on 6AM-6PM) was maintained.

### 2.4 Flow cytometry

Mice were euthanized via CO_2_ asphyxiation, after which whole blood, bone marrow, spleens, and brains were collected 7 and 30 DPI. Blood was collected with EDTA-lined syringes by cardiac puncture into 1.5 ml Eppendorf tubes, placed on ice, and processed as previously described (24). Red blood cells were lysed before centrifugation of remaining cells. Supernatant was removed and Fc receptors were blocked with anti-CD16/CD32 antibody. Cell pellets were incubated with the following antibody solution: Ly6G (FITC; BD Biosciences), Ly6c (PerCP-Cy5.5; Invitrogen), CD11b (APC; Invitrogen), CD3 (APC-Cy7; BD Biosciences), and B220 (PE-Cy7; BD Biosciences) for 15 minutes at room temperature. Cells were re-suspended in PBS for analysis.

Bone marrow was collected from the femur and flushed out using ice-cold PBS. Collected bone marrow was rinsed with PBS and filtered through a 70-μm cell strainer before being pelleted at 900 x g for 6 min. Cells were resuspended in PBS before being incubated in following antibody solution: Fc receptor block (anti-CD16/CD32), Ly6G (FITC), Ter119 (PE; BD Biosciences), Ly6c (PerCP-Cy5.5), CD11b (APC), and B220 (PE-Cy7) for 15 minutes at room temperature.

Spleens were collected immediately following CO_2_ asphyxiation and placed into PBS filled tubes on ice. Spleens were rinsed with PBS and filtered through a 70 -μm cell strainer before being pelleted at 900 x g for 6 min. Cells were resuspended in PBS before being incubated in following antibody solution: Fc receptor block (anti-CD16/CD32), Ly6G (FITC), Ter119 (PE), Ly6c (PerCP-Cy5.5), CD11b (APC), B220 (PE-Cy7), and CD3 (APC-Cy7) for 15 minutes at room temperature.

Brains were divided into ipsilateral and contralateral hemispheres before leukocyte isolation as previously described (24). Brains were homogenized into 5 mL of PBS in a glass Potter homogenizer before being transferred to a 15 mL conical and pelleted at 900 x g for 6 minutes. Following supernatant removal cell pellets were resuspended in 70% isotonic Percoll. A discontinuous Percoll density gradient was then applied in three layers: 50%, 35%, and 0% (PBS) isotonic Percoll. The gradient was centrifuged for 20 min at 2070 x g with low acceleration and brake. The layer containing fat debris and myelin was removed from all tubes and leukocytes were collected from the interphase between the 70% and 50% Percoll layers. The leukocyte layer was washed to remove any remaining Percoll, cells were pelleted at 900 × g for 6 min, and supernatant was removed. After cell isolation, Fc receptors were blocked with anti-CD16/CD32 antibody. Cells were washed and then incubated in the following antibody solution: Ly6c (PE; BD Biosciences), Ly6G (FITC), CD45 (PerCP-Cy5.5; BD Biosciences), CD11b (APC), CD3 (APC-Cy7), and B220 (PE-Cy7) for 15 minutes at room temperature. Cells were re-suspended in PBS for analysis. Previous studies have reported that viable cells isolated by Percoll density gradient yields >90% leukocytes (26, 27).

Single stain reference controls were used for spectral unmixing and autofluorescence removal. The flour-minus-one (FMO) method was used to assess non-specific binding and positive labeling for cell populations. Samples were run on the Cytek Aurora 3 laser system for spectral flow cytometry analysis. Data was analyzed using FlowJo software. Tissue collection was performed in two technical replicates (cohorts) for each group with (*N* = 6) 7 DPI and (*N* = 5-6) 30 DPI.

### 2.5 Fluorescence-activated cell sorting and RNA isolation

All mice were euthanized by CO_2_ asphyxiation and brains were collected 30 DPI. Brains were first bisected and placed into a stainless-steel coronal mouse brain matrix. Razor blades were placed 2mm apart in the matrix, perpendicular to midline and centered over the site of injury to collect a 2mm-thick coronal slice from the ipsilateral hemisphere. The slice was snap frozen in liquid nitrogen. CD11b^+^ myeloid cells were isolated from the remainder of the ipsilateral hemisphere via Percoll gradient, as previously described (12, 26). Briefly, tissue was homogenized using manual homogenizers, and the homogenate was centrifuged and resuspended in 70% Percoll (Sigma-Aldrich). A discontinuous Percoll density gradient was layered to collect CD11b^+^ cells. To isolate microglia from this cell layer, all cells were labeled were CD45 and CD11b antibodies and sorted on a Cytek Aurora cell sorter. CD11b^+^/CD45^Int^ microglia were collected, and RNA was extracted using PicoPure kit following manufacturer protocol (ThermoFisher). RNA was isolated from ipsilateral coronal brain slice tissue using Trizol following the Tri-Reagent protocol (Sigma-Aldrich). Tissue collection and RNA extraction were performed in three technical replicates (cohorts) for each group. RNA was extracted from N = 7–9 mice total per experimental group.

### 2.6 Statistical analysis

For righting times and flow cytometry, statistical analysis was completed within Prism 10.0.2 (GraphPad). For all experiments, sample sizes were determined based on previous studies (12, 18, 19). For righting time and flow cytometry data, 2-way analysis of variance (ANOVA) was utilized with injury (Sham or TBI) and sleep condition (Con or SF) as independent variables. Main effects of injury and condition, along with interaction effects, were considered. Tukey *post hoc* analysis was performed when interaction effects were detected in experiments. All comparisons with p < 0.05 were reported. For all experiments, researchers were blinded to animal group identification during data analysis.

### 2.7 RNA sequencing and analysis

RNA-Seq libraries were prepared using the Ovation SoLo RNA-Seq System, following manufacturer protocol (NuGEN). Libraries were sequenced on Illumina NovaSeq, single-end 100 base pair reads, 40 million reads per sample. Reads were then mapped to the mouse genome (assembly GRCm39) and gene-specific counts were generated using STAR version 2.6.0 (28). Statistically significant (q <0.05, |log_2_fold-change| > 2) differentially expressed transcripts were identified using DESeq2 (version 1.42.0). Protein-coding differentially expressed genes (DEGs) were analyzed using Enrichr (E. Y. 29–31) and ShinyGO (32).

## 3 Results

### 3.1 Post-injury SF does not significantly exaggerate peripheral immune cell populations at subacute or chronic timepoints

**Figure 1:**
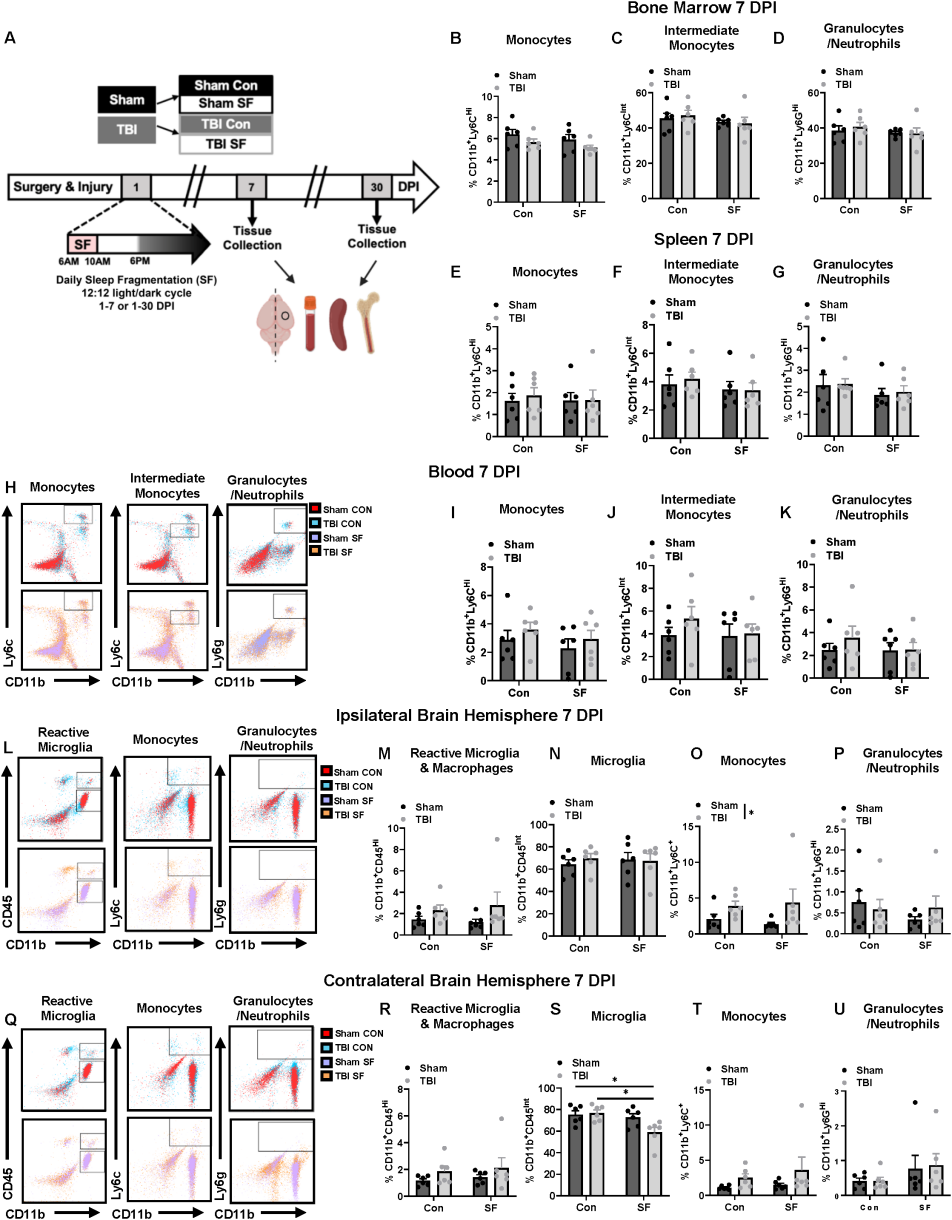
TBI transiently increases peripheral immune cell infiltration in the ipsilateral hemisphere 7 DPI that is not exaggerated by post-injury SF. **(A)** Mice received TBI or sham injury, followed by 7 or 30 consecutive days of daily sleep fragmentation or control conditions. Brain, blood, spleen, and bone marrow was collected at either timepoint for flow cytometry. Brains were bisected along midline and the hemispheres ipsilateral and contralateral to the injury site were separately processed. Markers were stained to look at cells of interest, including monocytes (CD11b^+^, Ly6C^Hi^; CD11b^+^, Ly6C^Int^) and granulocytes (CD11b^+^, Ly6G^Hi^) in the bone marrow **(B–D)** and spleen **(E–G)**. **(H)** Representative dot plots showing the gating strategy for identifying monocytes (CD11b^+^, LY6C^Hi^; CD11b^+^, Ly6C^Int^), and granulocytes (CD11b^+^, Ly6G^Hi^) in the ipsilateral hemisphere. **(I–K)** Quantification of cells of interest in the blood**. (L, Q)** Representative dot plots showing the gating strategy for identifying reactive microglia/macrophages (CD11b^+^, CD45^Hi^), monocytes (CD11b^+^, Ly6C^+^), and granulocytes (CD11b^+^, Ly6G^Hi^) in the ipsilateral hemisphere and contralateral hemisphere. Percentage of these cell populations were quantified within the ipsilateral **(M–P)** and contralateral **(R–U)** hemispheres. *N* = 6/group; error bars indicate SEM; * indicates (*p* < 0.05). DPI, days post-injury; SF, sleep fragmentation; SEM, standard error of the mean; TBI, traumatic brain injury. Portions of Panel **(A)** created in BioRender. Boland, R. (2025) https://BioRender.com/1edaezn.

Flow cytometry was used to define the distribution of peripheral immune cells following TBI and SF 7 and 30 DPI. Adult C57BL/6 mice received lateral FPI or sham injury, followed by 7 or 30 days of either control housing or SF housing, with daily mechanical SF from 6AM to 10AM. Peripheral tissues, including blood, spleen, and bone marrow, along with ipsilateral and contralateral brain were collected at both 7 and 30 DPI immediately following the last cycle of SF (**Figure 1A**). As expected, TBI caused significantly longer righting times than sham injury (**Supplementary Figure S1A**) (18–20). A posteriori analysis confirmed that righting time was similar between sham and brain injured mice that subsequently received control housing or SF (**Supplementary Figure S1A**).

Within the blood, bone marrow, and spleen, cells of interest included monocytes (CD11b^+^Ly6c^Hi^, CD11b^+^Ly6c^Int^), granulocytes (CD11b^+^Ly6G^Hi^), B cells (B220^Hi^), and red blood cells (CD11b^-^Ter119^+^). At 7 DPI, a similar distribution of peripheral immune cells was found in the bone marrow and spleen between experimental groups (**Figure 1**). However, there was a TBI-induced increase in monocytes (CD11b^+^Ly6c^+^) within the ipsilateral brain 7 DPI (Injury, *F*(1, 18) = 14.76 *p* < 0.05; **Figure 1O**). Other cells of interest in the brain included microglia populations defined as CD11b^+^CD45^Int^ and CD11b^+^CD45^Hi^. Notably, highly reactive microglia and peripheral macrophages are expected to be included in the CD11b^+^CD45^Hi^ population. There were no significant differences in reactive microglia/macrophages in either hemisphere 7 DPI. However, there was a significant decrease in CD11b^+^CD45^Int^ microglia in the contralateral hemisphere of TBI SF mice compared to Sham Controls and TBI Controls, which was not observed in the ipsilateral hemisphere (SF, *F*(1, 20) = 7.840, *p* < 0.05; Injury x SF, *F*(1, 20) = 4.470,*p* < 0.05; **Figure 1S**).

**Figure 2:**
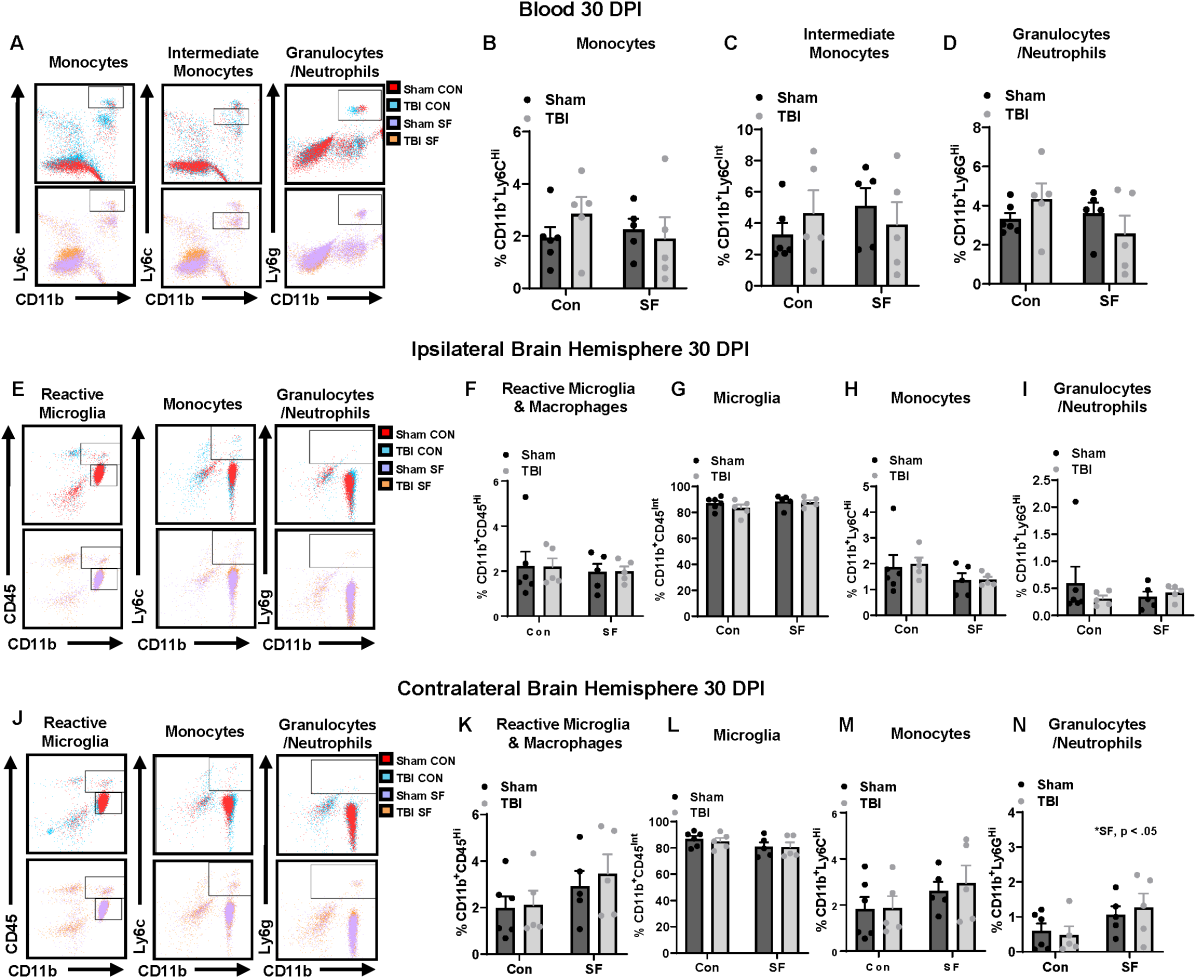
Peripheral immune cell populations were not significantly altered by TBI or SF 30 DPI. **(A, E, J)** Representative dot plots showing the gating strategy for identifying monocytes (CD11b^+^, Ly6C^Hi^; CD11b^+^, Ly6C^Int^), granulocytes (CD11b^+^, Ly6G^Hi^), reactive microglia/macrophages (CD11b^+^, CD45^Hi^) in blood **(A)**, ipsilateral hemisphere **(E)**, and contralateral hemisphere **(J)**. Cells of interest were quantified within the blood **(B–D)**, ipsilateral hemisphere **(F–I)**, and contralateral hemisphere **(K–N).***N* = 5-6/group; error bars indicate SEM; * indicates (*p* < 0.05). DPI, days post-injury; SF, sleep fragmentation; SEM, standard error of the mean; TBI, traumatic brain injury.

To determine the role of peripheral immune cells in the chronic immune response to TBI and SF, the same tissues and cell populations were analyzed 30 DPI. Here, TBI increased the presence of granulocytes and Ly6c^Int^ monocytes within the bone marrow (**Supplementary Figure S2**; Injury, *F*(1, 17) = 5.752, *p* < 0.05; **Supplementary Figure S2E**; Injury, *F*(1, 17) = 6.895, *p* < 0.05; **Supplementary Figure S2G**). Notably, this response was largely driven by brain injured animals in control housing. A similar distribution of peripheral immune cells was found in the spleen and blood between experimental groups 30 DPI. Within the brain, there were no significant differences in monocyte or microglia populations in either hemisphere chronically post-injury (**Figure 2**). This suggests that differences in microglia and monocytes 7 DPI represent transient changes driven by TBI and TBI-SF that resolve over time. Interestingly, there was a SF-driven increase in granulocyte populations 30 DPI, though this change was only observed in the contralateral hemisphere (SF, *F*(1, 17) = 4.942, *p* < 0.05; **Figure 2I**).

This comprehensive flow cytometric analysis confirms that post-injury SF does not exaggerate the peripheral immune response 7 and 30 DPI. Specifically, these analyses show no significant infiltration of peripheral monocytes into the brain due to TBI or SF at 30 DPI (**Figure 2**). These data also confirm no significant effects of injury or SF on proportion of microglia in the ipsilateral brain 30 DPI (**Figures 2F–H**). This is relevant because monocyte derived macrophages and highly reactive microglia express similar genes and proteins, making it difficult to distinguish the cell types with other conventional methods.

### 3.2 Using bulk RNA sequencing to analyze microglia-specific effects of TBI and SF on gene expression 30 DPI in the ipsilateral brain

**Figure 3:**
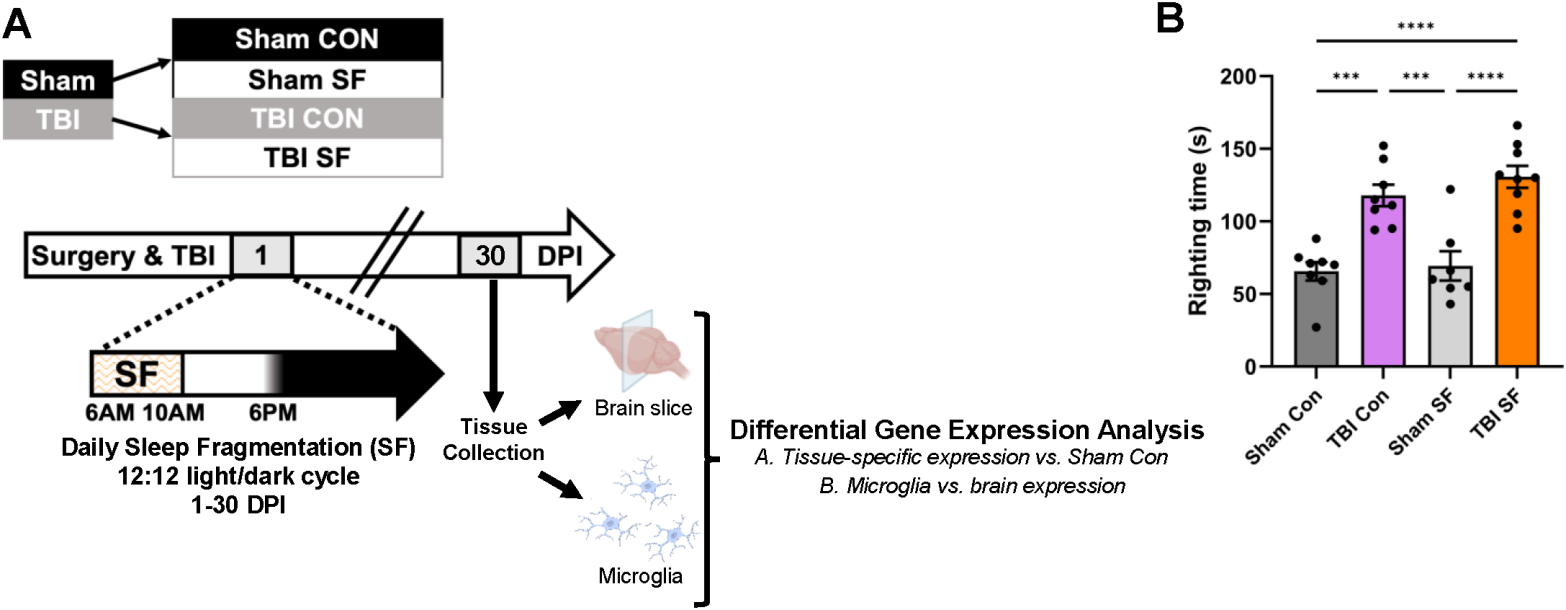
Differential gene expression analysis following post-injury sleep fragmentation. **(A)** Mice received TBI or sham injury, followed by 30 consecutive days of daily sleep fragmentation or control conditions. Tissue was collected at 30 DPI for gene expression analysis. A coronal brain slice was collected from the ipsilateral hemisphere as a representative sample of all brain cell types, and microglia were cell-sorted from the remainder of ipsilateral tissue. **(B)** Righting reflex was measured immediately following injury, and animals that received TBI had significantly longer time to right. *** indicates p < 0.001, **** indicates p < 0.0001 (2-way ANOVA, Tukey's multiple comparisons test). Portions of Panel **(A)** created in BioRender. Taylor, M. (2025) https://BioRender.com/0irwb18.

Previous data from our lab indicate persistent microglial activation and significantly increased pro-inflammatory gene expression in the ipsilateral cortex 30 DPI (19). We therefore hypothesized that the RNA profiles of microglia and other brain cells are uniquely dysregulated by TBI and post-injury SF, independent of peripheral immune cell infiltration. To test this, mice were exposed to either control housing or our established SF protocol for 30 days following sham injury or TBI. Consistent with previous studies, mice that received TBI had significantly longer time to right than sham mice (18, 20, 24) (**Figure 3B**). Brain tissue was collected 30 DPI, and we used RNA-sequencing to analyze DEGs of sorted microglia from the ipsilateral hemisphere and an ipsilateral coronal brain slice (**Figure 3A**). The primary goal was to identify the individual and combined effects of TBI and SF on gene expression, emphasizing the specific impact on microglia by comparison to coronal slice tissue, representative of multiple brain cell types. CD11b+/CD45^Int^ microglia were selected for these experiments to reduce the potential influence of monocyte derived macrophages on gene expression signatures. Additionally, we compared microglial gene expression directly to that of brain tissue to identify unique effects of TBI and SF in microglia compared to all ipsilateral brain cell types. For each tissue type, total RNA was isolated and sequenced, and transcriptomes for each experimental condition (TBI Con, TBI SF, and Sham SF) were compared to control (Sham Con) (**Figure 2A**). For analysis, we narrowed our scope to the top significantly dysregulated (q <0.05 and |log2fold-change| >2) differentially expressed genes (DEGs) in each experimental condition.

### 3.3 TBI and SF differentially affect olfaction-related genes in ipsilateral brain tissue 30 DPI

**Figure 4:**
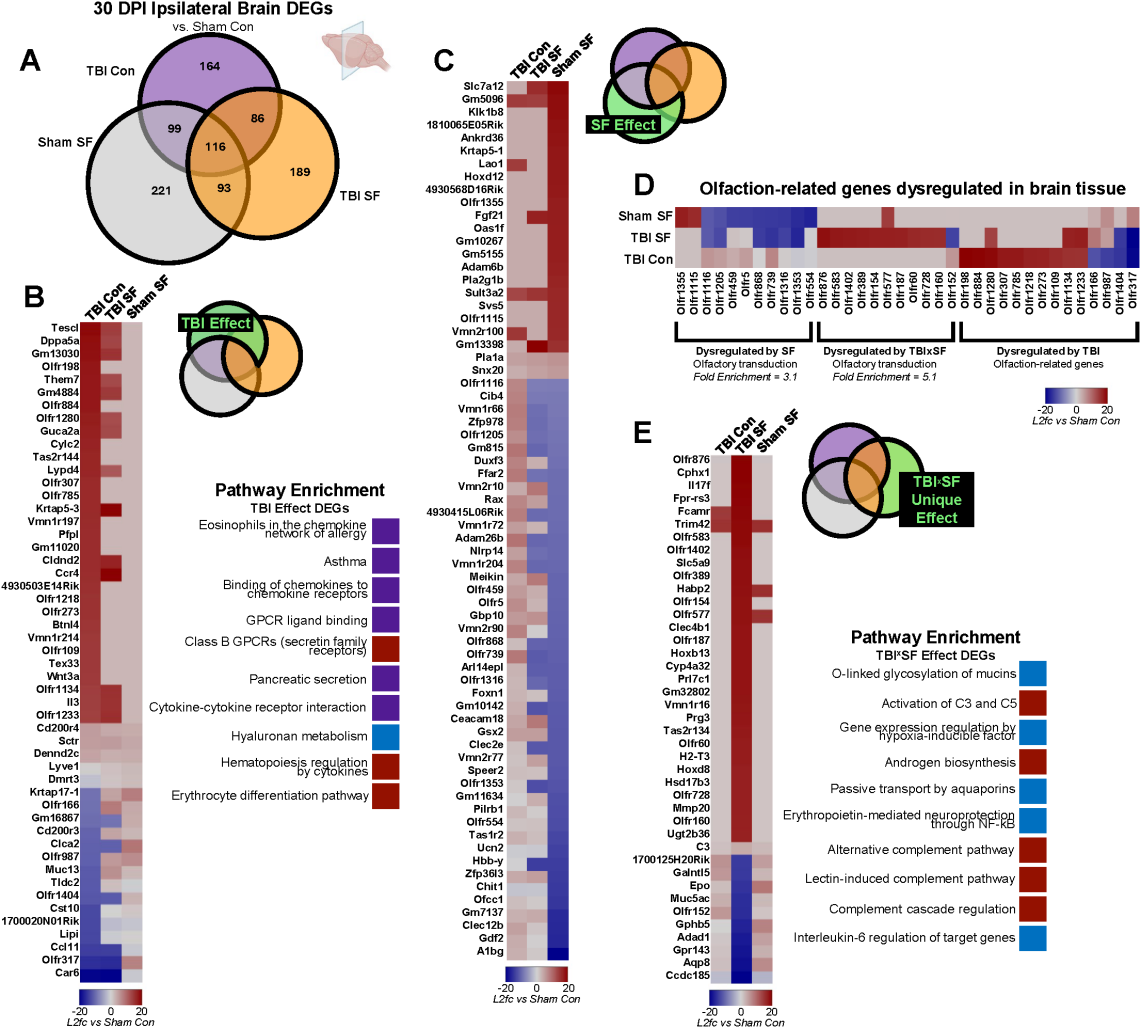
Effects of TBI and SF on ipsilateral brain gene expression. **(A)** DESeq2 was used to analyze differential expression in brain tissue, with sham control as a reference. Venn diagrams show the number of significant DEGs for each experimental condition. Results were filtered by q <0.05 and |log2fold-change| >2. **(B–E)** Independent and combined effects of TBI and SF on DEGs were determined. Heatmaps show significant DEGs, with cell color indicating log2fold-change value compared to Sham Con. Pathway enrichment analyses show top ten significant (p<0.05) pathway terms for each gene set. Red boxes indicate pathways corresponding to upregulated genes, blue boxes correspond to downregulated genes, and purple boxes indicate pathways with both up- and down-regulated genes. **(B)** Effect of TBI on gene expression was determined through analysis of genes significantly dysregulated in TBI Con group and TBI Con x TBI SF overlap, excluding overlap with Sham SF. **(C)** Effect of SF on gene expression in microglia and brain tissue was determined through analysis of genes significantly dysregulated in Sham SF group and Sham SF x TBI SF overlap, excluding overlap with TBI Con. **(D)** Heatmap shows olfaction-related genes significantly dysregulated by TBI, SF, and TBIxSF. **(E)** Effect of TBI SF on gene expression in microglia and brain tissue was determined through analysis of genes significantly dysregulated only in TBI SF group, excluding all overlap with Sham SF and TBI Con. Portions of Panel **(A)** created in BioRender. Taylor, M. (2025) https://BioRender.com/0irwb18.

We first investigated the effects of TBI and SF on gene expression in the ipsilateral brain (**Figure 4**). To identify effects of TBI, we focused on DEGs in the TBI Con group and genes dysregulated by both TBI SF and TBI Con, excluding all DEGs in the Sham SF group (**Figure 4B**). We identified a total of 250 DEGs affected by TBI in ipsilateral brain, 51 of which were protein-coding genes (**Figures 4A, B**). Gene set enrichment analysis revealed chemokine, allergy, and asthma-related pathways in these DEGs (**Figure 4B**). To determine the effects of SF, we analyzed genes dysregulated by Sham SF and TBI SF and excluded all genes dysregulated in the TBI Con group (**Figure 4C**). There were 314 DEGs affected by SF in ipsilateral brain, with 68 of these encoding for proteins (**Figure 4C**). Our gene set enrichment analysis identified significant pathway enrichment for olfactory transduction (KEGG database, 11 pathway genes), and no other pathway enrichment was found in this gene set. Interestingly, 9 out of the 11 olfaction-related genes were significantly downregulated (log2fold-change < -14, q <0.05) by SF. It is worth noting that many olfaction-related genes in brain tissue were significantly dysregulated by TBI, though this did not correspond to olfactory pathway categories in our enrichment analysis (**Figure 4D**). Enrichment analysis revealed that protein-coding DEGs from TBI SF brain tissue were also enriched for olfactory transduction (KEGG database, 11 pathway genes). 10 of the 11 genes were significantly upregulated, in contrast to the SF-induced downregulation of other olfaction-related genes. Similarly, TBI alone caused significant upregulation of another set of olfactory genes (**Figure 4D**). Together this suggests that TBI and SF have opposing effects on olfaction-related genes in the brain. SF causes significant downregulation of a set of olfactory genes, while TBI, with or without post-injury SF, upregulates an entirely different set of olfaction-related genes.

To determine how TBI and SF interact to affect gene expression in the ipsilateral brain, we focused only on those genes which are uniquely dysregulated in the TBI SF group and have no overlap with TBI Con or Sham SF (**Figure 4E**). This gene set comprised 189 total genes, including 41 protein-coding genes (**Figure 4E**). Several immune-related pathways were enriched in the protein-coding DEGs of TBI SF brain tissue, including the alternative complement pathway and NF-kB. IL-6 signaling was also influenced by TBI SF, namely through significant downregulation of the mucin gene *Muc5ac* (log2fold-change = -17.34), a direct target of IL-6. One of the most upregulated genes in TBI SF brains was *Il17f*, which encodes an IL-17 pro-inflammatory cytokine family protein (log2fold-change = 18.7 in TBI SF, q = 0.001), and this was not dysregulated by either TBI or SF alone (log2fold-change = 0 in TBI Con; log2fold-change = 0 in Sham SF). This suggests that some cytokine signaling pathways in the brain, including IL-17 and IL-6, are uniquely influenced by the combination of TBI and SF.

### 3.4 TBI and SF differentially dysregulate microglial steroid biosynthesis processes 30 DPI

**Figure 5:**
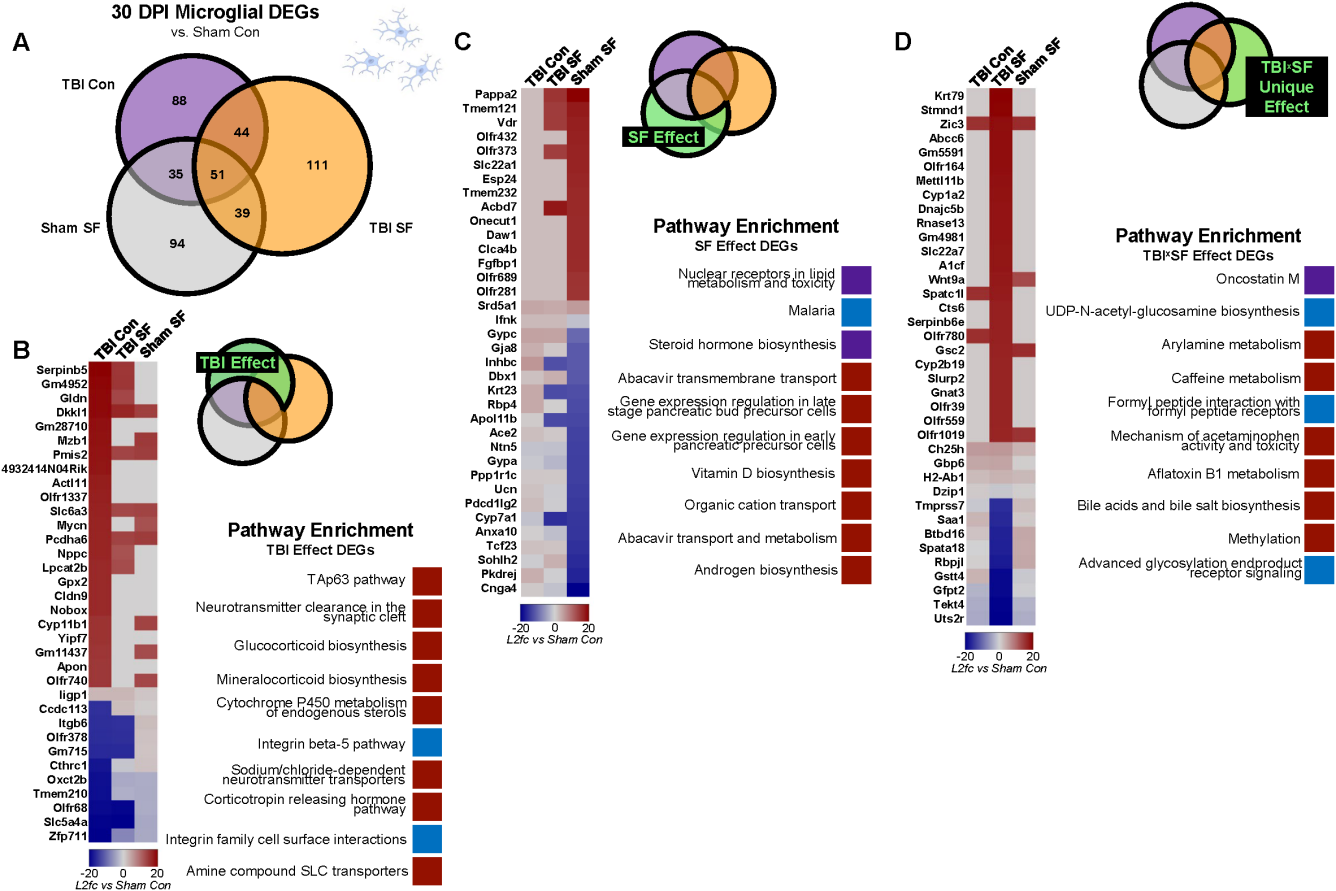
Effects of TBI and SF on ipsilateral microglia gene expression. **(A)** DESeq2 was used to analyze differential expression in microglia, with sham control as a reference. Venn diagrams show the number of significant DEGs for each experimental condition. Results were filtered by q <0.05 and |log2fold-change| >2. **(B–D)** Independent and combined effects of TBI and SF on DEGs were determined. Heatmaps show significant DEGs, with cell color indicating log2fold-change value compared to Sham Con. Pathway enrichment analyses show top ten significant (p<0.05) pathway terms for each gene set. Red boxes indicate pathways corresponding to upregulated genes, blue boxes correspond to downregulated genes, and purple boxes indicate pathways with both up- and down-regulated genes. **(B)** Effect of TBI on gene expression was determined through analysis of genes significantly dysregulated in TBI Con group and TBI Con x TBI SF overlap, excluding overlap with Sham SF. **(C)** Effect of SF on gene expression in microglia and brain tissue was determined through analysis of genes significantly dysregulated in Sham SF group and Sham SF x TBI SF overlap, excluding overlap with TBI Con. **(D)** Effect of TBI SF on gene expression in microglia and brain tissue was determined through analysis of genes significantly dysregulated only in TBI SF group, excluding all overlap with Sham SF and TBI Con. Portions of Panel **(A)** created in BioRender. Taylor, M. (2025) https://BioRender.com/0irwb18.

We next analyzed the effects of TBI and SF specifically on gene expression in ipsilateral microglia (**Figure 5A**). We identified 132 DEGs, including 34 protein-coding genes, with significant TBI-induced expression changes in microglia (**Figure 5B**). Enrichment for the TAp63 tumor suppression pathway, as well as several pathways related to corticosteroid synthesis and function, were identified within these genes (**Figure 5B**). A total of 133 DEGS, including 36 protein coding genes, were significantly dysregulated by SF in microglia. Enrichment analysis suggests disruption of a variety of pathways, including lipid metabolism and transmembrane transport. Interestingly, steroid hormone biosynthesis was one of the top pathways dysregulated by SF (**Figure 5C**). This is similar to the enrichment categories for microglial TBI DEGs, but through dysregulation of different genes. *Cyp11b1*, encoding a steroid hydroxylase, is significantly (log2fold-change = 16.46) upregulated in microglia of TBI Controls (**Figure 5B**). A different hydroxylase gene, *Cyp7a1*, is dysregulated by SF in microglia (**Figure 5C**). Interestingly, *Cyp7a1* is significantly downregulated in both Sham SF and TBI SF groups (log2fold-change = -21.2 and -22.11, respectively). This suggests that distinct steroid biosynthesis pathways are differentially dysregulated by TBI and SF in microglia.

To explore the combined effect of TBI and SF on microglial genes, we next focused on genes uniquely dysregulated in the TBI SF group. There were a total of 111 microglial DEGs in this group, 38 of which were protein-coding genes. Enrichment analysis highlighted several immune-related processes, including iscosanoid transport and lymphocyte chemotaxis (**Figure 5D**). Several genes related to the oncostatin M pathway, involved in IL-6 signaling, are dysregulated in microglia (33). This includes upregulation of *Ch25h*, *Cyp1a2*, and *Slc22a7*, and downregulation of *Saa1* (**Figure 5D**). These data suggest differential effects of post-TBI SF on immune pathways, including IL-6 signaling, in the microglia compared to the whole brain.

### 3.5 Microglia exhibit TBI-induced dysregulation of innate immune and acetylcholine receptor pathways compared to whole brain 30 DPI

Importantly, the brain tissue analyzed in these experiments contains multiple cell types, including microglia. Thus, to more closely dissect how microglial gene expression is influenced by TBI and post-TBI SF, and to understand how microglial dysregulation contributes to changes in the brain, we conducted a separate analysis to compare microglia expression directly to that of the ipsilateral brain (**Figure 3A**). This was accomplished by setting the RNA profile of brain tissue for each experimental condition as the baseline for comparison and measuring microglia differential expression relative to the brain. This resulted in four sets of DEGs, representing all four experimental conditions including Sham Control (**Supplementary Figure S3A**). To control for differential gene expression that could be due to any aspect of surgery or isoflurane exposure, we subtracted any genes that overlapped with the Sham Control group, a total of 14,011 DEGs (**Supplementary Figure S3B**). We focused the rest of our analysis on significant DEGs in the TBI Control, TBI SF, and Sham SF groups that did not have any overlap with Sham Control (**Supplementary Figure S3C**).

**Figure 6:**
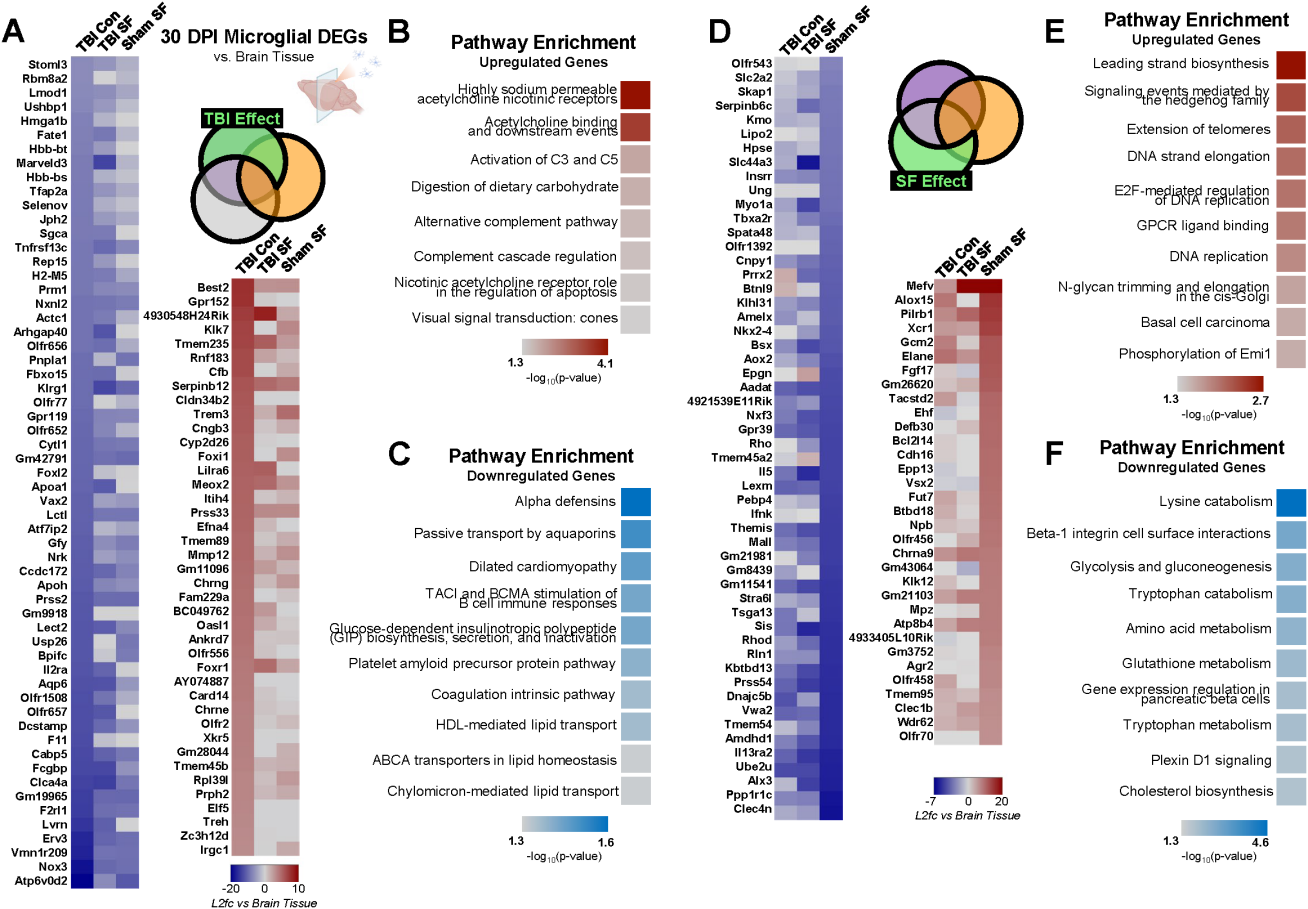
Influence of TBI and SF on microglial gene expression compared to whole brain. **(A)** Effect of TBI on microglial gene expression compared to whole brain was determined through analysis of genes significantly (q <0.05, |log2fold-change| > 2) dysregulated in TBI Con group and TBI Con x TBI SF overlap, excluding all overlap with Sham SF. Heatmaps show significant protein-coding DEGs, with cell color indicating log2fold-change value of each gene compared to brain tissue of the same experimental condition **(B).** Heatmaps represent subset of protein-coding DEGs - the top 59 downregulated (log2fold-change < -3) and top 41 upregulated (log2fold-change >3) genes are shown. **(B, C)** Gene set enrichment analysis was performed on all significant (q <0.05, |log2fold-change| > 2) DEGs, and top pathway enrichment categories for upregulated and downregulated genes are shown. **(D)** Effect of SF on microglial gene expression compared to whole brain was determined through analysis of genes significantly (q <0.05, |log2fold-change| > 2) dysregulated in Sham SF group and Sham SF x TBI SF overlap, excluding all overlap with TBI Con. Heatmaps show significant DEGs, with cell color indicating log2fold-change value of each gene compared to brain tissue of the same experimental condition. The top 54 downregulated (log2fold-change < -3) and top 33 upregulated (log2fold-change >3) genes are shown. **(E, F)** Gene set enrichment analysis was performed on all significant DEGs, and top pathway enrichment categories for upregulated and downregulated genes are shown. Portions of Panel **(A)** created in BioRender. Taylor, M. (2025) https://BioRender.com/0irwb18.

We identified 924 DEGs significantly (q <0.05, |log2fold-change| > 2) dysregulated by TBI, either in the TBI Con group or in the overlap between TBI Con and TBI SF (**Supplementary Figure S3C**; **Figure 6A**; heatmaps show subset of DEGs - top 59 downregulated (log2fold-change < -3) and top 41 upregulated (log2fold-change >3) genes). A total of 306 of these DEGs were protein-coding genes. Of these, 132 are upregulated and 174 are downregulated. Gene set enrichment analysis of downregulated DEGs revealed enrichment for a variety of pathways, including immune-related alpha defensins and B cell stimulation. Upregulated genes corresponded to other immune-related pathways, namely the alternative complement pathway through upregulation of *Cfb* (log2fold-change = 5.4 in TBI Con, **Figures 6B, C**). Additionally, enrichment analysis suggests upregulation of acetylcholine receptor processes. *Chrng* and *Chrne*, both of which encode for different subunits of the acetylcholine receptor protein, are significantly upregulated in TBI Control microglia relative to whole brain tissue (log2fold-change = 4.09 and 3.5, respectively).

There were a total of 857 DEGs significantly dysregulated by SF, and 251 of these were protein-coding genes (**Supplementary Figure S3C**; **Figure 6D**; heatmaps show subset of DEGs - top 54 downregulated (log2fold-change < -3) and top 33 upregulated (log2fold-change >3) genes). 91 protein-coding DEGs were upregulated and 160 downregulated by SF. Gene set enrichment analysis revealed enrichment for several metabolism- and catabolism-related pathways in downregulated genes. Upregulated genes were enriched for multiple regulatory DNA pathways, including DNA strand elongation and DNA replication (**Figures 6E, F**).

### 3.6 Post-TBI SF specifically affects steroidogenesis and cell-to-cell communication pathways in microglia compared to whole brain 30 DPI

**Figure 7:**
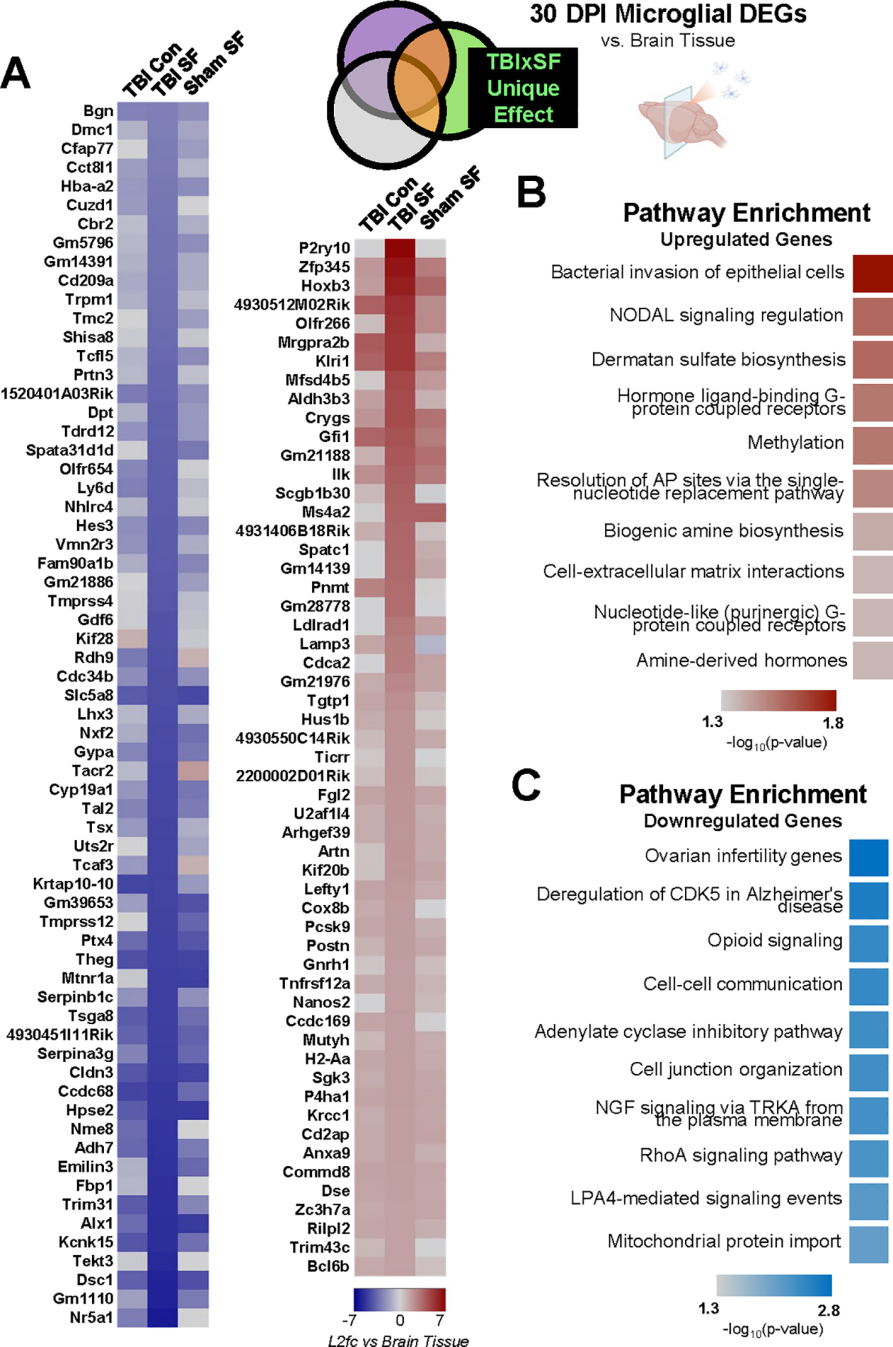
Gene expression changes in microglia compared to rest of brain following post-TBI SF. **(A)** Effect of TBI SF on microglial gene expression compared to whole brain was determined through analysis of genes significantly (q <0.05, |log2fold-change| > 2) dysregulated only in TBI SF group, excluding all overlap with Sham SF and TBI Con. Heatmaps show significant DEGs, with cell color indicating log2fold-change value of each gene compared to brain tissue of the same experimental condition. Of the 165 downregulated genes, the top 65 (log2fold-change<-3) are shown. All 55 upregulated (log2fold-change>2) are shown. **(B, C)** Gene set enrichment analysis was performed on all significant (q <0.05, |log2fold-change| > 2) DEGs, and top pathway enrichment categories for upregulated and downregulated genes are shown. Portions of Panel **(A)** created in BioRender. Taylor, M. (2025) https://BioRender.com/0irwb18.

A total of 682 DEGs were significantly dysregulated by the unique combination of TBI and SF in microglia compared to ipsilateral brain (**Supplementary Figure S3C**, **Figure 7A**). 220 of these DEGs encode proteins, with 55 upregulated and 165 downregulated genes. Within both up- and downregulated DEGs, enrichment analysis indicates dysregulation of a variety of distinct pathways, including two G-protein coupled receptor pathways in upregulated genes and an Alzheimer’s-related CDK5 pathway in downregulated genes (**Figures 7B, C**). Surprisingly, the most significantly enriched pathway among downregulated genes corresponded to ovarian infertility. This included significant downregulation of *Nr5a1*, *Dmc1*, and *Cyp19a1* (log2fold-change = -6.25, -3.08, and -4.83, respectively). Interestingly, *Nr5a1* (steroidogenic factor 1) and *Cyp19a1* (aromatase) are both implicated in steroid hormone synthesis. This suggests suppression of steroidogenesis pathways in microglia, relative to the whole brain, after post-TBI SF, in contrast to the upregulation of other steroid-related pathways we found in microglia after TBI alone (**Figure 5B**). Interestingly, cell surface interaction pathways appear in both up- and downregulated DEGs (**Figure 6C**). The majority of genes corresponding to these pathways are downregulated, including *Cdh5*, *Cldn3*, *Pard6a*, *Cdh24*, and *Mapk12* (**Figure 6B**). This downregulated gene set suggests dysregulation of cell-cell communication in microglia compared to the ipsilateral brain, and could reflect structural and functional changes undergone by microglia when TBI is combined with post-injury SF.

## 4 Discussion

Previous work from our lab showed that both 7 and 30 days of post-injury mechanical SF induces microglial morphological restructuring, increasing neuroinflammation and impairing behavioral outcomes compared to TBI alone (19, 24). Blood-brain-barrier (BBB) disruption after TBI contributes to peripheral immune cell infiltration to the brain (9, 21, 22, 34).Reactive glia can also recruit peripheral immune cells to the central nervous system to help respond to injury by releasing inflammatory cytokines and chemokines and assisting with debris clearance (9, 34, 35). Therefore, peripheral immune cells play a critical and possibly distinct role in shaping the neuroimmune response to TBI as well as post-injury SF. In this study, we investigated the independent and combined effects of TBI and post-injury SF on the neuroimmune landscape. We analyzed the distribution of peripheral immune cells, resident macrophages, and microglia using flow cytometry. We also measured transcriptional changes in a coronal brain slice and sorted microglia using bulk RNA sequencing. We hypothesized that post-TBI SF would have a robust influence on the ipsilateral transcriptome 30 DPI. Indeed, we found that SF and TBI both induce significant dysregulation of a variety of transcripts in the brain and specifically in microglia. Further, the combination of TBI with post-injury SF alters the microglia and brain transcriptome significantly, compared to TBI alone.

Several findings warrant further discussion. Previous preclinical studies consistently demonstrate that peripheral immune cells are actively responding to brain injury during the acute phase (i.e. ~1–3 DPI) of recovery (36, 37). Detection of peripheral immune cell trafficking and brain infiltration declines in the subacute and chronic phases of recovery (38). Here, there were no significant differences in monocyte or granulocyte populations in any of the peripheral tissues 7 DPI. TBI caused a significant increase in ipsilateral cortical monocytes 7 DPI, though this resolved by 30 DPI. There was a TBI-induced increase in granulocytes within the bone marrow 30 DPI, but there were no changes in peripheral immune cell populations within the blood or spleen 30 DPI. While these results could reflect chronic alterations in post-injury myelopoiesis, further assessment is needed to understand this chronic shift in the bone marrow. SF caused an increase in granulocytes and neutrophils in the contralateral brain 30 DPI, but this was not detected in the ipsilateral hemisphere. Multiple pre-clinical studies across different injury models demonstrate robust immune cell infiltration early (4 hours - 3DPI) in the response to TBI (39–41). This may be due in part to robust BBB disruption that is seen acutely post-injury in pre-clinical models (42–45). However, similar studies show no significant differences in circulating immune cell populations in the blood or infiltrating immune cells in the brain 7 DPI (46–48). While the peripheral immune response at chronic post-injury timepoints after FPI has not been fully characterized, there is support that peripheral immune cell populations are reduced in the chronic phase post-injury in other TBI models (49–51). Research from clinical studies has demonstrated that BBB disruption can be long lasting (52–54). However, these current findings suggest that BBB disruption may subside following the acute phase of injury, as shown in previous literature (55–57). Therefore, the results are in accordance with existing literature and show that SF following TBI does not exaggerate or prolong the peripheral immune response between the sub-acute and chronic phases of recovery.

Our previous studies demonstrated that TBI and SF increased percent area of Iba17 and 30 DPI in the injured cortex (18, 19, 58), which could result from microglial proliferation. There were no differences in CD11b^+^CD45^Hi^ or CD11b^+^CD45^Int^ brain populations in the injured cortex 30 DPI. This observation could tell us that the microglial response to TBI and SF involves morphological restructuring and not exclusive expansion of the microglia cell population. Flow cytometry may mask region specific differences in microglia that are better visualized with techniques such as immunofluorescence. Nevertheless, flow cytometry is superior to immunofluorescence in distinguishing monocytes, monocyte derived macrophages, and microglia, which have similar expression of key surface proteins such as CD11b and CD45. We expect that monocyte derived macrophages as well as highly reactive microglia are included in the CD11b^+^CD45^Hi^ cell population. Therefore, to avoid the potential influence of infiltrating monocytes, we selected the CD11b^+^CD45^Int^ brain population for subsequent bulk RNA sequencing analysis. Our flow cytometry data revealed a significant increase in monocyte populations in the ipsilateral hemisphere driven by TBI 7 DPI. However, there were no significant changes between our conditions in immune cell populations in the ipsilateral hemisphere 30 DPI. These data demonstrate that peripheral immune cell infiltration 7 DPI was transient and resolved by the chronic phase post-injury. Furthermore, we found no significant differences in the proportion of CD11b^+^CD45^Int^ cells between experimental groups 30 DPI, which ensured consistency in the type of cells that were collected for gene expression analysis. Thus, sequencing 30 DPI represents the ideal timepoint to understand transcriptomic changes within microglia in the chronic phase of TBI.

TBI and SF induced robust dysregulation of brain and microglial RNA. In brain tissue, olfactory receptor genes contributed to a large portion of protein-coding DEGs in each experimental condition. These receptors are largely expressed on olfactory sensory neurons in the nasal cavity and nasal epithelium, where they function in chemosensation as G protein-coupled receptors. However, mounting evidence in mice and humans demonstrates their expression and function outside of the olfactory system, including other neuron types in the brain (59–61). Here, we present further evidence of olfactory receptor expression in non-olfactory tissue. Interestingly, clinical evidence indicates that alteration or loss of olfaction is an early symptom of TBI, and multiple preclinical studies have investigated the link between TBI and olfaction (62–65). Controlled cortical impact TBI in mice caused microglia-mediated inflammation resulting in neuronal deficits in the olfactory bulb (62). Neurons from the olfactory bulb project to other brain regions to relay sensory information, and impaired olfactory neuron function could impact other neuron types through this network. Additionally, recent evidence from clinical serum samples showed release of extracellular vesicles (EVs) containing mRNA of several olfactory receptors from injured neurons after TBI, suggesting broad upregulation of expression of olfactory receptor genes (66). Our findings here support TBI-induced upregulation of these receptors, and our data suggests differential effects of TBI and SF on olfactory receptor expression in the brain. The effects of SF alone on olfaction are understudied. Our results suggest that contrary to the effects of TBI, SF downregulated a distinct set of olfactory genes. It will be necessary for future studies to determine how these gene sets influence each other, and whether TBI and SF interact to affect olfactory function.

While much of our analysis was focused on the differential effects of TBI and SF, we also identified some interesting overlap. Within microglia, we found that TBI and SF both affected steroid synthesis pathways, but through dysregulation of different genes. For example, two distinct hydroxylase genes, *Cyp11b1* and *Cyp7a1*, were differentially dysregulated by TBI and SF. *Cyp11b1* was significantly upregulated by TBI, while *Cyp7a1* was downregulated by SF. *Cyp11b1* encodes 11β-hydroxylase, an enzyme required for glucocorticoid and mineralocorticoid production (67). Clinical evidence demonstrates that TBI stimulates immediate activation of the HPA axis stress response, elevating serum cortisol levels within the first 24 hours following injury (68, 69). By contrast, *Cyp7a1* encodes cholesterol 7α-hydroxylase, primarily studied for its role in bile acid synthesis in the liver (70, 71). However, cholesterol homeostasis is important to brain function, and similar cholesterol hydroxylases have been shown to play a key role in these pathways (72). Cholesterol dysregulation has been shown to impact microglia function, and recent evidence suggests that cholesterol homeostasis is critical for microglia-mediated repair in the contexts of Alzheimer’s disease and multiple sclerosis (73, 74). The magnitude of downregulation (log2fold-change -21.2 in Sham SF and -22.1 in TBI SF) of *Cyp7a1* in microglia after SF could reflect a significant loss of function. Additionally, in our direct comparison of microglia gene expression to that of the brain, we identified other steroidogenesis genes that were significantly downregulated following post-injury SF. These data suggest that TBI induces upregulation of steroid pathway genes in microglia, while SF downregulates other steroid pathways. When TBI is followed by post-injury SF, further downregulation of additional steroidogenesis genes occurs in microglia. Together, this suggests that both TBI and SF affect steroid biosynthesis, but likely through dysregulation of different pathways.

TBI induces an initial inflammatory response in multiple cell types, including microglia (75, 76). The precise signaling pathways involved vary from cell to cell and can depend on a variety of factors including age, sex, and injury severity. The immune effects of TBI become even more complex as the injury progresses to longer time points and chronic neuroinflammation persists (4, 77). In our analysis, we identified multiple immune-related genes that were only significantly dysregulated by the combination of TBI and SF and were unaffected by either TBI or SF alone. One of the most upregulated genes in TBI SF brains was *Il17f*, which encodes a pro-inflammatory cytokine mainly secreted by helper T cells (78). We did not detect T cell populations in our flow cytometry analyses of the brain, but this data suggests increased expression of *Il17f* RNA by one or more cell types in the ipsilateral hemisphere.

Pathway enrichment analyses suggest that IL-6 signaling was influenced by post-TBI SF in both the brain and in microglia. IL-6 is a key inflammatory cytokine released by multiple cell types, including microglia and neurons, to promote neuron survival and repair after TBI (79, 80). Previous studies demonstrate an acute increase in IL-6 after TBI, with significantly elevated gene and protein levels detectable hours after TBI (81, 82). Due to the low physiological levels and rapid release after injury, IL-6 has been explored as a potential biomarker for TBI, and elevated levels are correlated with worsened outcome after injury (83, 84). Here, we did not detect any significant changes in *Il6* gene expression 30 days after TBI or SF. However, in brain tissue, we found significant downregulation of a direct target of IL-6, *Muc5ac*. This could reflect upstream changes in IL-6 signaling. Additionally, TBI SF caused dysregulation of several components of the oncostatin M (OSM) signaling pathway in microglia, including significant upregulation of *Ch25h*, *Cyp1a2*, and *Slc22a7*, and downregulation of *Saa1*. OSM is a member of the IL-6 cytokine family and has previously been shown to play a neuroprotective role in spinal cord injury and multiple sclerosis (MS) (85, 86). Previous studies have shown that microglia produce OSM in response to inflammatory signaling, and elevated OSM is associated with neurological diseases including MS and glioblastoma (87–89). Our results suggest alterations in the microglial OSM signaling pathway after post-TBI SF, which could reflect changes in OSM production.

When we compared microglia gene expression directly to brain tissue, many of the top DEGs were related to cell-cell interaction and communication processes. Most of these corresponded to downregulated genes, including cadherins (*Cdh5*, *Cdh24*) and claudin 3 (*Cldn3*), membrane proteins important for maintaining cell-cell adhesion and morphology. Previous studies have demonstrated downregulation of *Cldn3* after hypoxia and ischemia, causing blood brain barrier damage, and *Cldn3* is also implicated in impaired oligodendrocyte migration and function in white matter disease (90, 91). Less is known about the functions of *Cdh5* and *Cdh24*, though their expression has been reported in multiple brain regions (92, 93). The specific role of these membrane proteins in microglia has not been determined. Our findings here suggest that genes encoding multiple of these cell-cell interaction proteins are significantly downregulated in microglia compared to other brain cells after post-injury SF.

### 4.1 Conclusions

The goal of this study was twofold: (1) To investigate how TBI and post-TBI SF influence the peripheral and microglia immune response 7 and 30 DPI and (2) to analyze how SF alters the effects of TBI on whole brain and microglia gene expression at 30 DPI. While TBI and post-TBI SF induced transient changes in microglia and monocyte populations 7 DPI, modest effects of TBI and SF were found 30 DPI. Specifically, the proportion of peripheral immune cells and microglia were similar between experimental groups within the brain 30 DPI. Our transcriptional analysis revealed that TBI and SF differentially influence multiple biological processes in the brain and microglia, including olfaction and steroidogenesis pathways. Importantly, we found that TBI and SF combine to induce robust gene dysregulation not influenced by TBI or SF alone. Our results shed light on potential molecular mechanisms of microglia-mediated dysfunction after post-injury SF. It is worth noting that specific sub-clusters of microglia were not identified in this bulk RNA sequencing analysis. Bulk RNA sequencing provides insight to broad trends in microglia expression, this approach may not be sufficient to capture the heterogeneity of all microglia phenotypes. Therefore, higher resolution techniques such as single cell RNA sequencing are necessary to determine the effects of lateral fluid percussion TBI and SF on specific microglia sub clusters. While this study included both male and female mice, more work is needed to determine if post-injury SF influences gene signatures in a sex dependent manner. Together, these data align with our previous work demonstrating that environmental sleep disruption after TBI significantly influences the neuroimmune response to injury. Continued effort is needed to determine how post-injury sleep disruption shapes the microglial response over time. Broadly, these results also lend support to interventions aimed at mitigating chronic neuroinflammation after TBI and improving long term outcome.

## Data Availability

Original RNA sequencing datasets are publicly available through the NCBI Sequence Read Archive. This data can be found here: https://www.ncbi.nlm.nih.gov/bioproject/PRJNA1369438.

## Supplementary material

Supplementary Figure 1 TBI and SF do not significantly alter red blood or B cells 7 DPI**. (A)** TBI significantly increased righting time compared to sham (main effect TBI, *p* < 0.05). **(B, C)** Quantification of red blood cells in the bone marrow **(B)** and spleen **(C)**. **(D-H)** Quantification of B cells in the brain **(D, E)**, blood **(F)**, bone marrow **(G)**, and spleen **(H)**.

Supplementary Figure 2 TBI and SF do not significantly influence immune cell populations in the brain or periphery 30 DPI. **(A)** TBI significantly increased righting time compared to sham (main effect TBI, *p* < 0.05). **(B, C)** Quantification of B cells in the brain and blood **(D)**. **(E–I)** Quantification of granulocytes, monocytes, red blood cells, and B cells within the bone marrow. **(J–N)** Quantification of granulocytes, monocytes, red blood cells, and B cells within the spleen.

Supplementary Figure 3 Comparing microglia gene expression to brain tissue. DESeq2 was used to analyze differential expression in microglia and brain tissue, with brain tissue as a reference. Venn diagrams show significant DEGs in microglia compared to brain tissue for each experimental condition **(A)**. To control for effects of surgery and anesthesia, any overlap with Sham Con group was subtracted **(B)** from TBI Con, TBI SF, and Sham SF DEGs **(C)**. Results were filtered by q <0.05 and |log2fold-change| >2.
