# Supplementary figures and images for: Traumatic brain injury and post-injury sleep fragmentation differentially alter the microglial transcriptome
Source: Front Immunol. 2026 Jan 5;16:1689773. doi: 10.3389/fimmu.2025.1689773 (PMC12812616; doi:10.3389/fimmu.2025.1689773)

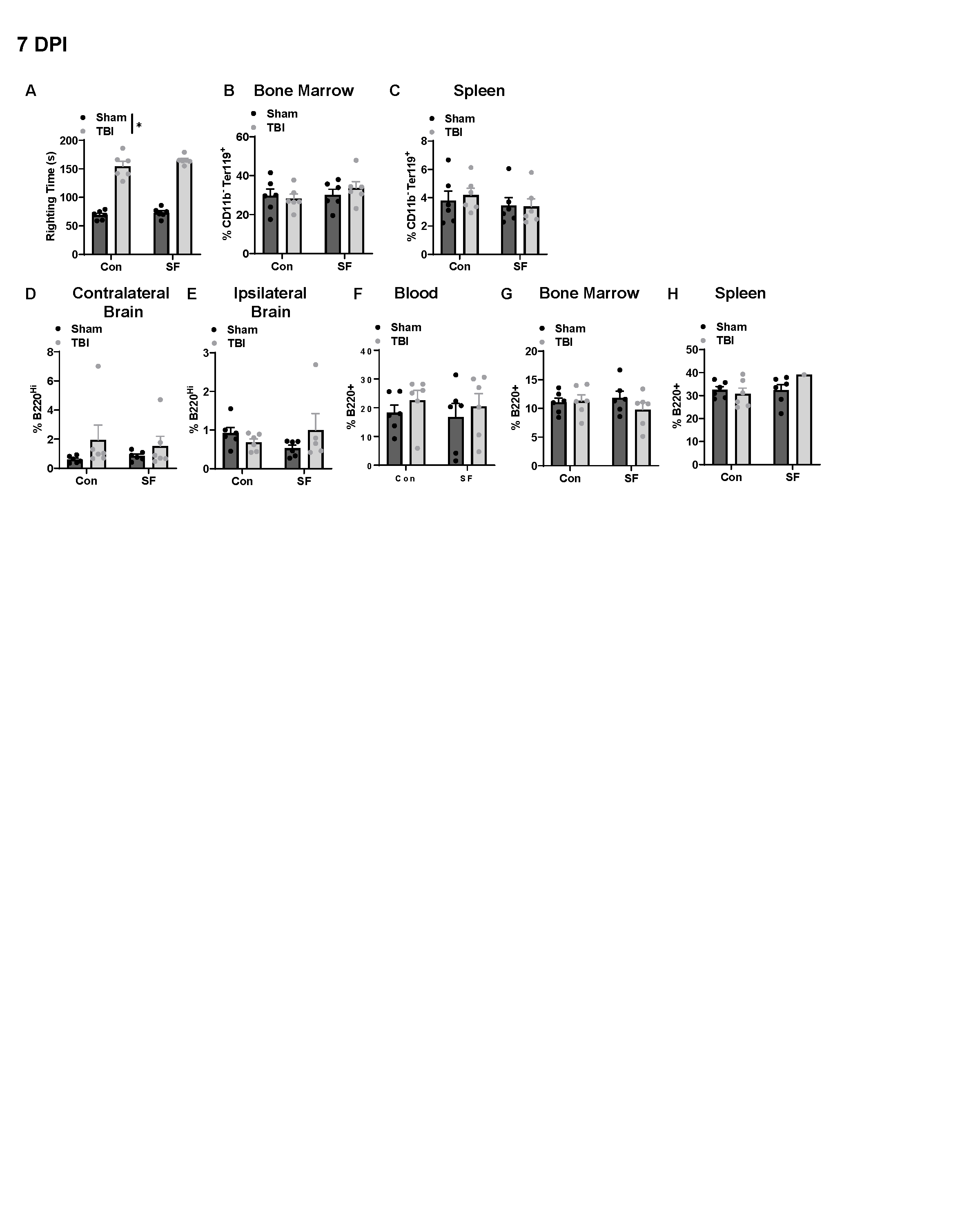

Supplement: Supplementary Figure 1 — TBI and SF do not significantly alter red blood or B cells 7 DPI. (A) TBI significantly increased righting time compared to sham (main effect TBI, p < 0.05). (B, C) Quantification of red blood cells in the bone marrow (B) and spleen (C). (D-H) Quantification of B cells in the brain (D, E), blood (F), bone marrow (G), and spleen (H). [file Image1.tif]

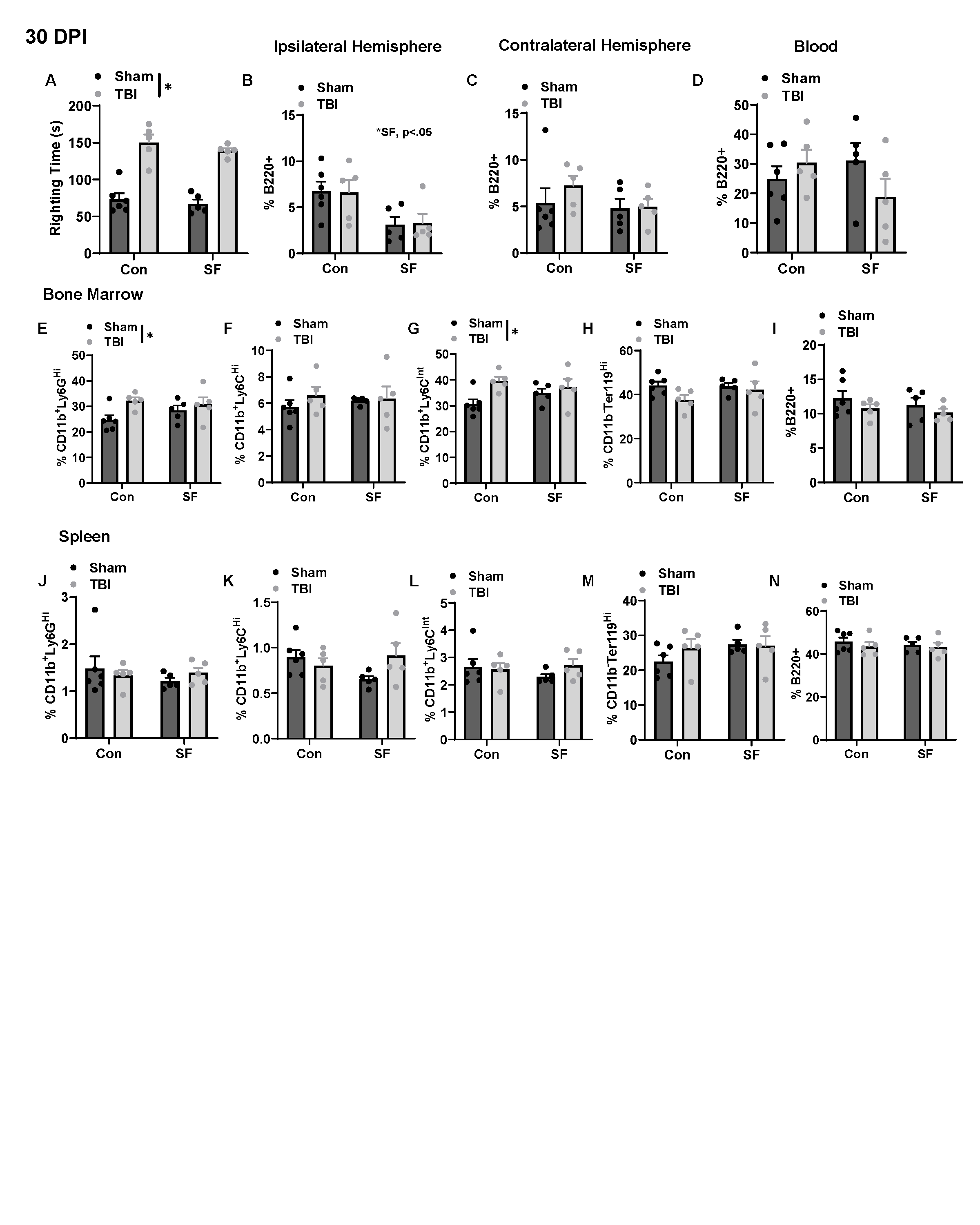

Supplement: Supplementary Figure 2 — TBI and SF do not significantly influence immune cell populations in the brain or periphery 30 DPI. (A) TBI significantly increased righting time compared to sham (main effect TBI, p < 0.05). (B, C) Quantification of B cells in the brain and blood (D). (E–I) Quantification of granulocytes, monocytes, red blood cells, and B cells within the bone marrow. (J–N) Quantification of granulocytes, monocytes, red blood cells, and B cells within the spleen. [file Image2.tif]

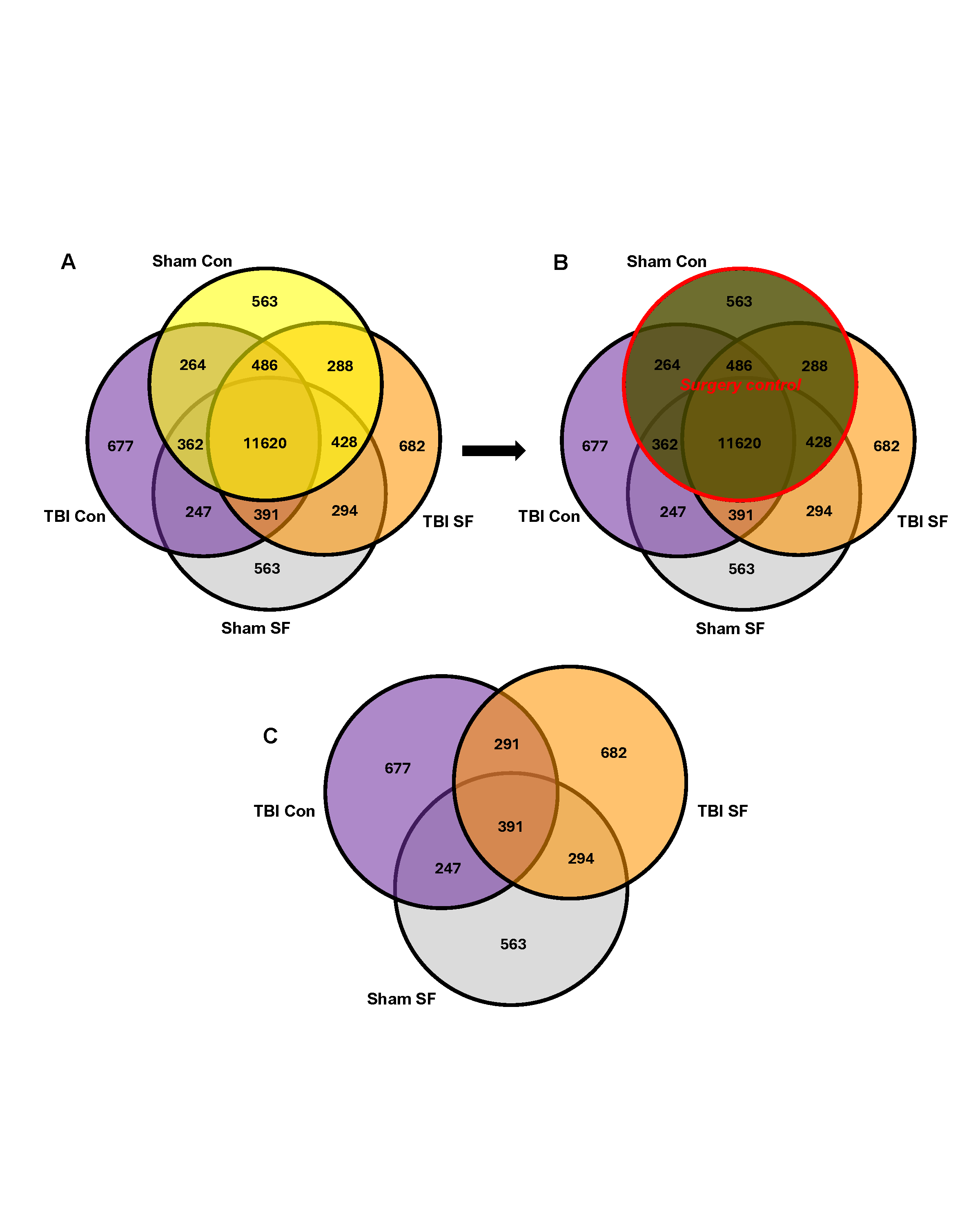

Supplement: Supplementary Figure 3 — Comparing microglia gene expression to brain tissue. DESeq2 was used to analyze differential expression in microglia and brain tissue, with brain tissue as a reference. Venn diagrams show significant DEGs in microglia compared to brain tissue for each experimental condition (A). To control for effects of surgery and anesthesia, any overlap with Sham Con group was subtracted (B) from TBI Con, TBI SF, and Sham SF DEGs (C). Results were filtered by q <0.05 and |log2fold-change| >2. [file Image3.tif]
